# Power of the Dissolution Test in Distinguishing a Change in Dosage Form Critical Quality Attributes

**DOI:** 10.1208/s12249-018-1197-7

**Published:** 2018-10-22

**Authors:** Vivian A. Gray

**Affiliations:** V. A. Gray Consulting, Inc., Hockessin, Delaware USA

**Keywords:** dissolution, critical material attributes, critical product properties, discriminatory power, *in vivo* performance

## Abstract

For a dissolution method to be considered relevant to *in vivo* performance, the dissolution data profiles should show discrimination or meaningful change when there is a change in critical material attributes (CMAs) and critical product properties (CPPs). The dissolution test has been shown repeatedly to have the power to distinguish between significant changes in active pharmaceutical ingredient (API), formulation, and process that relate to the release mechanism of the *in vivo* performance. Examples will be discussed in the literature where the effects of formulation, drug substance, and manufacturing variables have been measured by dissolution testing. There will be a suggested plan on how to develop and challenge a discriminating method that may be utilized for regulatory purposes. A brief review of other challenges and considerations regarding discriminatory dissolution testing is presented**.**

## INTRODUCTION

Following the history of the dissolution test, the USP became the first organization to show an interest in dissolution testing by creating a USP-National Formulary Panel to examine bioavailability and ways to test release mechanisms to provide some, albeit *in vitro*, assurance for drug effectiveness. This Panel recommended the dissolution test, using the rotating basket apparatus, be placed in monographs in USP. During the 1970s, there were 12 official dissolution tests using baskets in USP monographs. The paddle apparatus followed shortly thereafter. In the early 1980s, the USP Expert Committee on Biopharmaceutics proposed a single-point method: 75% Q in 45 min with water as a medium.

In 1997, testing using profiles came into prominence through an FDA guidance document (1) where FDA first started to require dissolution profiles rather than just a single time point test. These profiles showed the dissolution rate over the entire time period of the test. Typically, the measured time points are 15, 30, 45, and 60 min for immediate release dosage forms such as tablets or capsules. The profile time points were not used as specifications but as information about the product’s release rate.

As the test evolved, it became a critical tool for establishing biowaivers for highly soluble compounds (2). Here is the first example of dissolution showing or substituting for *in vivo* performance. There have been many papers and guidances which now promote the use and development of discriminating dissolution specifications. Discriminating dissolution specifications is defined in Abend *et al*. (3) as “A set of in vitro dissolution testing conditions that, along with the acceptance criterion, are able to differentiate drug products manufactured under target conditions vs products that are intentionally manufactured with meaningful variations … for the relevant manufacturing variables …”. In this context, the discriminating method would generate “clinically relevant specifications” (4,5). Developing a method that is relevant to *in vivo* performance has been the latest challenge from industry, academia, and regulatory agencies, and this is a driving force to develop methods that can show the power to distinguish between significant changes in CMA and CPP that relate to the release mechanism variables that are linked to the *in vivo* performance. There are continuing efforts through the EMA “Reflections” paper (6), FDA and EMA guidances on biowaivers (2,7), the ongoing biopharmaceutical project OrBiTo (http://www.orbitproject.eu) and, in the realm of harmonization, with ICH M9 (8) to promote discriminatory dissolution methods.

### Part 1: Discriminating Dissolution Testing Power

Discriminatory dissolution methods are keys to providing a regulatory method that is meaningful for the release of drug into the body of the patient. The term discriminating method, for purposes of this review, is one that will distinguish changes and is sensitive to the variables of the drug substance, formulation, and manufacturing process. Suarez *et al*. (5) elaborates that the dissolution test is unique as it measures the effect of formulation and physical properties of the drug substance on the rate of drug solubility. Burgess *et al*. (9) discussed how *in vitro* methods should be designed on the basis of *in vivo* release mechanisms. Yu *et al*. (10) explains how the concepts of quality by design (QbD) provide a systematic approach to linkage of product quality to the desired clinical performance. The identification of the CMA and CPP is a critical part of the product development and will be critical knowledge for the development of a discriminating dissolution method.

A paper written by members of the AAPS (American Association of Pharmaceutical scientists) *In Vitro* Release and Dissolution Testing Focus Group (11) anticipated in 2009 the utility of the dissolution test to distinguish among variables. The article provided several papers that showed the ability of the test to detect differences in such critical parameters as disintegrant types, packaging, storage temperature and humidity, hardness, polymorphs, and lubricants. In this paper, more of these types of studies will be provided.

### Part 2: Examples of the Dissolution Test Showing Change with Variations on Different CMA and CPP

The critical quality attributes (CQAs) of a drug product may include assay, content uniformity, degradation, and importantly to this topic, drug release (10). The CMA and CPP are aspects of that CQA for dissolution of the oral drug product.

Review of the literature shows that there is a continuing effort to determine how changes in CMA and CPP will also demonstrate a change in the dissolution rate. A selection of a few will be highlighted in this section of the paper.

Xia *et al*. (12) investigated the effect of crystal size on the dissolution of the poorly soluble drug, nitrendipine. Five different particles sizes (200 nm, 620 nm, 2.7 μm, 4.1 μm, and 20.2 μm) were evaluated in simulated intestinal media in the fasted state (FaSSIF). The dissolution rate of nitrendipine was significantly increased by the reduction in particle size with the FaSSIF media proving to be very useful in the discriminating dissolution method.

An evaluation of the influence of different processing methods on release of 5-aminasolicyclic acid from a matrix system is described in Korbely *et al*. (13). The matrix system was controlled by water-insoluble polymers and the different processing methods included, i.e., direct compression, wet granulation with water, or wet granulation with aqueous dispersions. The dissolution showed an increase with the wet granulation with water and a decrease was observed with direct compression. The dissolution media was 6.8 pH phosphate buffer using USP Apparatus 2 at 50 rpm.

The effect of compression pressure on the dissolution of cefuroxime axetil tablets was measured by Nanjwade *et al*. (14). Their studies showed that there was a sudden drop in release rate using higher compression pressure and hardness. The explanation was that the dissolution was influenced by the compact binding properties of discrete particles of cefuroxime axetil and the excipients. The dissolution media was 0.07 N HCl using USP Apparatus 2 at 55 rpm.

A study by Gökçe *et al*. (15) showed the effect of the tablet shape on the release rate of metronidazole lipid matrix tablets. The two different shapes were cylinder and hexagonal, and the study also included investigation of the surface area/volume effects on the lipid matrix. The conclusion was that the type of lipid matrix and geometric shape influenced the diffusion and release mechanism. The dissolution media was 0.1 N HCl using USP Apparatus 1 at 100 rpm.

Zhao *et al*. (16) evaluated the impact of amounts of certain excipients on the dissolution rate of formulations of acetaminophen and aspirin. The work used several typical excipients (disintegrants), croscarmellose sodium, crospovidone, sodium starch glycolate, and surfactant sodium lauryl sulfate (SLS). The results showed that crospovidone gave slower release rate in aspirin and use of SLS gave a longer disintegration time. The release of aspirin increased with the SLS despite the longer disintegration time. The dissolution media for acetaminophen was 5.8 pH phosphate buffer using the paddle at 50 rpm, and the method for aspirin was using pH 4.5 acetate buffer and the basket at 50 rpm.

The effect of different grades of HPMC and Eudragit on the drug release of doxofylline matrix tablets was evaluated by Panda *et al*. (17). The product was manufactured using wet granulation using various grades and ratios to optimize the dissolution profile. Using the *f*2 calculations to compare the various combinations of these CMA, the ideal combination was chosen. The dissolution method used USP Apparatus 2 with 2 h in simulated gastric fluid and the rest of the time in simulated intestinal fluid.

This last work by Narang *et al*. is provided because it provides in-depth information on the effect of excipient interactions with solid dosage form stability (18). The article supports the discussion of physical instability and the effects on dissolution rate with numerous references. A look at these references supports the power of the dissolution test to show change in CPP and importantly the change in dissolution rate of products over the self-life. There is also an excellent discussion of the role of water (moisture) on the stability of dosage properties and in particular dissolution rates.

### Part 3: Suggested Plan for Challenging Methods for Discriminating Power

The first step is the development of a discriminatory method. Rarely are varied lots available at this time so usually the challenge to the discriminating ability happens after a method is developed and validated. Additionally, the product may not be in its final iteration. This does pose a problem of having a method that may not be discriminating or even the best for the final product. The development of the final method may turn out to need some additional method development work to achieve discriminatory power.

The key to developing a robust discriminating method is to first follow the principles of a good method which typically are a distinctive gradual profile with appropriate time points and achieving over 85% dissolved, moderate to low variability, limited artifacts, and usually a media that is representative of the target site. Though no universal media is suggested there have been additives and fine tuning of media composition to assist in achieving better physiological relevance (19). The USP General Informational Chapter <1092> Dissolution Procedure: Development and Validation is a premier resource for developing a suitable method (20). Once an appropriate method is developed, it is always prudent to have an alternative method as a backup if the selected method shows to not be suitable.

The next challenge is to determine how and if variations on the dosage form can be manufactured. This is a very practical matter as resources may be limited. However, earlier exploratory batches or formulations (that have hopefully been retained) may offer some useful variations. For convenience the chosen lot to test and compare with variables will be called here the Selected Lot (SL). Ideally, there should be identified two or three variables, each, for the drug substance, formulation, and manufacturing process. In Yu (21) and Yu *et al*. (10), there are very helpful listings of CMA and CPP. In Dickerson *et al*. (22), there is an in-depth treatment and guidance to applying dissolution testing to assessing relevant variables and is highly recommended reading.

In two EMA guidances (6,7), there are sections that give advice on ways to demonstrate discriminatory power of dissolution methods. The charge is to manufacture a batch that varies a parameter. The suggestion is to vary parameters around ± 10–20% change of the SL parameter (5,20). One caution is to be sure the mechanism for release is not altered. In some cases, it is necessary to go beyond the ± 20% ranges stated above to see discriminatory power, in this case, the information is still relevant if there are supporting *in vivo* data to suggest this is the outer limits of bioequivalence. The dissolution data is collected and the profiles plotted and examined to see if a change in the dissolution rate could be matched and quantitated to the changes of higher (faster) and lower (slower) parameters.

When selecting the CMA of the drug substance, particle size is critical to dissolution rate and is of paramount importance as a variation. Others could include the surface area, crystal structure, and polymorphs. The drug substance could have slightly different characteristics depending on the manufacturing process, solvent used, age, packaging, and even the API manufacturing vendor.

The oral drug product has a variety of CPP, especially with modified release formulations. For example, parameters of coating thickness, porosity, excipient ratio, grade and purity, MW cutoff of polymers, *etc.*, could be examined. Immediate release formulations also have several important CPP, e.g., disintegrant type and level, magnesium stearate levels, binder level, and granule disintegration.

Manufacturing parameters in batch records offer a plethora of CPP ideas. Going over the step by step process of the dosage form manufacturing process is an exercise that could be illuminating and guide to selecting changes in a setting, speed, temperature, timing, addition order, *etc.* Dickerson *et al*. (22) provides for immediate release products BCS Class 2, a useful list of 11 “potential failure modes.” These include as the highest risk failure modes as (1) changes in drug substance particle size, (2) failure to control granulation end point (overgranulation), (3) increased level of binder, and (4) decreased level of disintegrant. Mechanical properties of the pharmaceutical materials, for example, brittleness, viscoelasticity, toughness, and hardness, should be considered (21).

Once the variations are manufactured, the testing can be a design of experiment (DoE) with a testing matrix or a one-variable-at-a-time. If the SL has moderate to high variably, then at least the testing of 6–12 dosage units is needed.

The SL on stability in different conditions or even open dish, with or without desiccant will provide important information on the discriminatory power of the method.

Determining if the dissolution rate of product variations is actually different goes back to the assessment of the test showing or picking up change. Some changes may be subtle or barely measurable, others dramatically different. Reporting all the data challenging the discriminatory power is the best approach as this shows what was tried and also demonstrates due diligence in trying to understand the dosage form along with evaluating the method for discriminatory power. To make a statement as to whether the dissolution method shows change in a particular parameter is to start with a graphical representation of the profiles (with error bars) of the SL and the upper and lower variations. A comparison can be made visually from the graph along with other comparison tools. The comparison tools for this particular task are not prescribed in any guidance but labs use several options: the *f*2 comparison equation, criteria in USP Chapter <1092> Dissolution Procedure: Development and Validation for intermediate precision, or other statistical tools (20). Most importantly is to show all the comparison dissolution profiles. This data will be part of the method development report. The entire exercise of performing these comparisons ultimately shows due diligence and also an understanding of the product and concepts of QbD.

The last step is determining a Q value that is meaningful, and, in context with *in vivo* performance. The prospect of an overly discriminating method may occur if that *in vivo* link is not established. The dissolution method should not only be discriminating but also, if possible, biopredictive.

The search is to determine what may have an effect on the dissolution rate, hence the release mechanism, possibly leading to a change *in vivo* performance. Identification of the rate limiting steps in the absorption process that can be linked to *in vivo* performance is of great importance. This linkage may be through an *in vitro in vivo* relationship, *in vitro* or *in vivo* correlation, and/or simulations (3). Lack of dissolution data linking the critical quality attributes to bioavailability and bioequivalence *in vivo* may lead to design spaces that are not acceptable (23).

## OTHER CONSIDERATIONS

A discriminatory method alone, though useful and better than a method that lacks this capability, is still less than ideal due to the lack of *in vivo* linkage. However, in a recent official guidance, it is stated that for highly soluble drug substances formulated in IR dosage forms there is no requirement to show discriminatory ability (24).

As we delve into discriminatory methods, there exits many challenges. Linking meaningful differences and *in vivo* performance is the critical challenge during method development as there is the potential problem of being overly discriminating. This may create a situation that points to certain processes or formulation variables that do not have quantitative relevance to absorption and may impose unnecessary restrictions on the manufacturing process (3).

A meaningful variant according to the EMA “Reflections” paper (6) may be aligned with BCS properties. However, the BCS property of drug solubility for example, is not necessarily a CPP, because it cannot be varied unless there are formulation modifiers. What constitutes a meaningful variant when dissolution testing is involved requires the in-depth knowledge of the release mechanism. This knowledge may or may not be completely known or understood, but the scientists, as part of the QbD concept, should strive to achieve and apply this understanding.

In some methods there is an *in vivo* link such as IVIVC or IVIVR, whereas in others the *in vitro* methods are supported by physiologically based pharmacokinetic (PBPK) modeling rather than *in vivo* parameters. There is an increased awareness of the utility of PBPK or mechanistic IVIVC modeling (25) along with many recent publications. Pepin *et al*. (26) showed how the dissolution rate could be justified using absorption PBPK modeling. This modeling tool was able to demonstrate that the proposed dissolution specifications were within the anticipated bioequivalence region. The use of *in vitro* and *in silico* was proposed by Ibarra *et al*. as an approach to screen product performance and target specific formulations for bioequivalence assessment (27). Other characteristics of the API (e.g., permeability) can indeed complicate matters when it comes to *in vitro* testing. With molecules of negligible solubility, aspects such as diffusion and erosion come into play. Lin, *et al*. (28) demonstrated in a study that the relationship between the dissolution test and *in vivo* outcome is complex and dependent on the characteristics of the drug molecule, product design, and the dissolution method conditions. The interplay between *in vitro* release and *in vivo* absorption has not been well understood and a generalized link has not been attempted.

A case study presented by Vuletić *et al*. develops a clinically relevant *in vitro* method along with investigating the pharmacokinetic response of formulation variants (29). The end result is to be able to develop an IVIVC model that would produce the prediction of absorption properties by testing the dissolution profiles and pharmacokinetic properties. This study was especially interesting as an IVIVC on an immediate release BCS class 2 compound was developed.

## CONCLUSION

The need for a discriminatory method is paramount from directives of the regulatory agencies. Through examples in the literature, it has been shown that the dissolution method provides the tool of showing difference among changes in the CMA and CPP. A suggested, practical approach has been discussed. There are ongoing efforts to strengthen the linkage to *in vivo* by use of improved media and PBPK.

The dissolution test has the potential and, in many cases, is that powerful tool that alerts that there may be bioinequivalent batches produced, therefore avoiding patients receiving drugs that are not fully efficacious.

## References

[1] Dissolution testing of immediate-release solid oral dosage forms. Guidance for Industry; U.S. Department of Health and Human Services, Food and Drug Administration, Center for Drug Evaluation and Research (CDER), Silver Spring, MD; 1997.

[2] Waiver of in vivo bioavailability and bioequivalence studies for immediate-release solid oral dosage forms based on a biopharmaceutics classification system. Guidance for Industry; U.S. Department of Health and Human Services, Food and Drug Administration, Center for Drug Evaluation and Research (CDER), Silver Spring, MD; 2017.

[3] Abend A, Heimbach T, Cohen M, Kesisoglou F, Pipen X, Suarez-Sharp S (2018). Dissolution and translational modeling strategies enabling patient-centric drug product development: the M-CERSI workshop summary report. AAPS J.

[4] Marroum PJ (2012). Clinically relevant dissolution methods and specifications. Am Pharm Rev.

[5] Suarez S, Marroum P, Hughes M. In: Shargel L, Yu A, editors. Chapter 15, Biopharmaceutic considerations in drug product design and in vitro drug product performance, Applied biopharmaceutics and pharmacokinetics. 7th ed: McGraw-Hill Education; 2016.

[6] Reflections paper on the dissolution specification for generic solid oral immediate release products with systemic action. European Medicines Agency. London, UK; 2017.

[7] Guideline on quality of oral modified release products. European Medicines Agency. London, UK; 2014.

[8] Biopharmaceutics Classification System-Based Biowaivers M9. http://www.ich.org. Verified on August 23, 2018.

[9] Burgess DJ, Hussain AS, Ingallinera TS, Chen M-L (2002). Assuring quality and performance of sustained and controlled release parenterals: AAPS workshop report, co-sponsored by FDA and USP. Pharm Res.

[10] Yu LX, Amidon G, Khan MA, Hoag SW, Polli J, Raju GK, Woodcock J (2014). Understanding pharmaceutical quality by design. AAPS J.

[11] Gray Vivian, Kelly Gregg, Xia Min, Butler Chris, Thomas Saji, Mayock Stephen (2009). The Science of USP 1 and 2 Dissolution: Present Challenges and Future Relevance. Pharmaceutical Research.

[12] Xia D, Cui F, Piao H, Cun D, Piao H, Jiang Y, Ouyang M, Quan P (2010). Effect of crystal size on the in vitro dissolution and oral absorption of nitrendipine in rats. Pharm Res.

[13] Korbely A, Kelemen A, Kasa P, Pintye-hodi K (2012). Effects of processing on the release profiles of matrix systems containing 5-aminosalicyclic acid. AAPS PharmSciTech.

[14] Nanjwade B, Ali S, Nanjwade V, Manvi F. Effect of compression pressure on dissolution and solid-state characterization of cefuroxime axetil. Journal of analytical and bioanalytical techniques. Open Access. 2010. 10.4172/2155-9872.1000112.

[15] Gökçe E, Ozyanzici M, Ertan G (2009). The effect of geometric shape on the release of properties of metronidazole from lipid matrix tablets. J Biomed Nanotechnol.

[16] Zhao J, Koo O, Pan D, Wu Y, Morkhade D, Rana S, Saha P, Marin A (2017). The impact of disintegrant type, surfactant and API properties on the processability and performance of roller compacted formulations of acetaminophen and aspirin. AAPS J.

[17] Panda N, Reddy V, Reddy G, Panda K (2015). Effect of different grades of hpmc and eudragit on drug release profile of doxofylline sustained release matrix tablets and IVIVC studies. Int Res J Pharm.

[18] Narang A, Desai D, Badawy S (2012). Impact of excipient interactions on solid dosage form stability. Pharm Res.

[19] Bou-Chacra N, Curo Melo KJ, Cordova Morales IA, Stippler ES, Kesisolgou F, Yazdanian M, *et al*. Evolution of choice of solubility and dissolution media after two decades of biopharmaceutical classification system. AAPS J. 2017;19(4). 10.1208/s/122248-017-0085-5. 10.1208/s12248-017-0085-5 28516359

[20] USP 40–NF 35, The dissolution procedure: development and validation <1092> USP: Rockville, MD; 2017.

[21] Yu LX (2008). Pharmaceutical quality by design: product and process development understanding, and control. Pharm Res.

[22] Dickerson PA, Lee WW, Stott PW, Townsend AI, Smart JP, Ghahramani P, Hammett T, Billet L, Behn S, Gibb R, Abrahamsson B (2008). Clinical relevance of dissolution testing in quality by design. AAPS J.

[23] Fotaki Nikoletta, Gray Vivian, Krämer Johannes, Diaz Dorys, Flanagan Talia, Grove Geoffrey (2016). Dissolution Highlights from the 2015 AAPS Annual Meeting in Orlando. Dissolution Technologies.

[24] Dissolution testing and acceptance criteria for immediate-release solid oral dosage form drug products containing high solubility drug substances. Guidance for Industry; U.S. Department of Health and Human Services, Food and Drug Administration, Center for Drug Evaluation and Research (CDER), Silver Spring, MD; 2018.

[25] Zhang X, Duan J, Kesisoglou F, Novakovic J, Amidon GL, Jamei M, Lukacova V, Eissing T, Tsakalozou E, Zhao L, Lionberger R (2017). Mechanistic oral absorption modeling and simulation for formulation development and bioequivalence evaluation: report of an FDA public workshop. CPT Pharmacometr Syst Pharmacol.

[26] Pepin XJH, Flanagan TR, Holt DJ, Eidelman A, Treacy D, Rowlings CE (2016). Justification of drug product dissolution rate and drug substance particle size specifications based on absorption PBPK modeling for lesinurad immediate release tablets. Mol Pharm.

[27] Ibarra M, Valiante C, Sopeña P, Schiavo A, Lorier M, Vázquez M, Fagiolino P (2018). Integration of in vitro biorelevant dissolution and in silico PBPK model of carvedilol to predict bioequivalence of oral drug products. Eur J Pharm Sci.

[28] Lin Zhongqiang, Zhou Deliang, Hoag Stephen, Qiu Yihong (2016). Influence of Drug Properties and Formulation on In Vitro Drug Release and Biowaiver Regulation of Oral Extended Release Dosage Forms. The AAPS Journal.

[29] Vuletić L, Khan MZI, Špoljarić D, Radić M, Cetina-Čižmek B, Filipović-Grčić J (2018). Development of a clinically relevant dissolution method for metaxalone immediate release formulations based on an IVIVC model. Pharm Res.

